# Supplementary material for: Light-to-Heat Converting ECM-Mimetic Nanofiber Scaffolds for Neuronal Differentiation and Neurite Outgrowth Guidance
Source: Nanomaterials (Basel). 2022 Jun 23;12(13):2166. doi: 10.3390/nano12132166 (PMC9268234; doi:10.3390/nano12132166)
Supplement: Supplementary file 1 [file nanomaterials-12-02166-s001.zip › nanomaterials-1747849-supplementary.pdf]

nanomaterials

Supplementary Materials

Light-to-Heat Converting ECM-Mimetic Nanofiber Scaffolds for Neuronal Differentiation and Neurite Outgrowth Guidance

Olga Y. Antonova *, Olga Y. Kochetkova and Igor L. Kanev

Institute of Theoretical and Experimental Biophysics, Russian Academy of Sciences, Pushchino, 142290 Moscow, Russia; o.y.kochetkova@gmail.com (O.Y.K.); 4kanev@gmail.com (I.L.K.)
* Correspondence: olga.antonova.iteb@gmail.com; Tel.: +7-9152622583

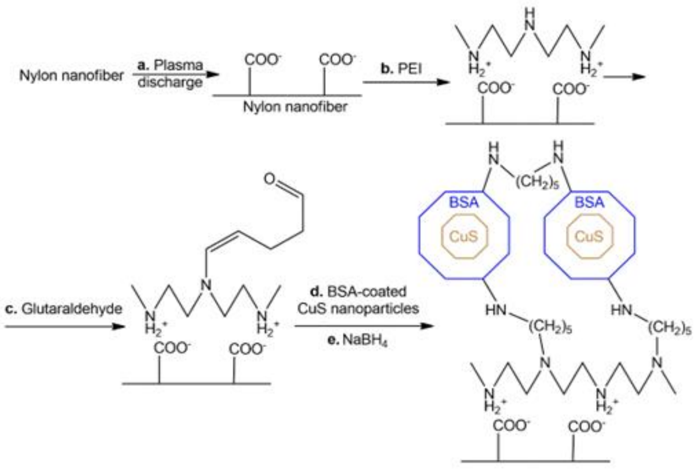

Figure S1. Schematic representation of CB-NPs immobilization on nylon AU fibers. **a.** Plasma pretreatment, 5 s. **b.** Branched PEI (25 kDa) 2% solution in water, 30 min, washing with water. **c.** Glutaraldehyde vapors, 30 min, washing with water. **d.** 1 mg/ml CB-NPs in 0.1 M HEPES, pH 7.0, 30 min, washing with 0.1 M HEPES. **e.** 1% sodium borohydride water solution, 15 min, washing with water.

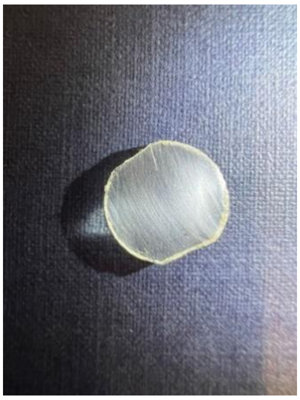

Figure S2. Image of the AU-In scaffold.

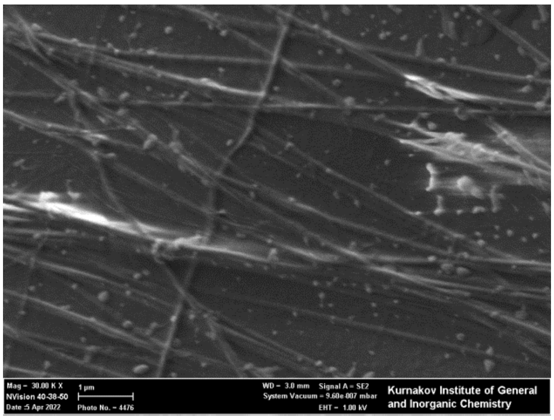

Figure S3. SEM image of the AU-Coat scaffold after incubation in aqueous PBS at 37C for a month.
